# Control of Green Mold and Sour Rot in Mandarins by Postharvest Application of Natamycin and an Allium Extract

**DOI:** 10.3390/plants13233428

**Published:** 2024-12-06

**Authors:** Elena Pérez Faggiani, Gerónimo Fernandez, Mariángeles Cocco, Mauricio Sbres, Oribe Blanco, Joanna Lado

**Affiliations:** 1 Programa Nacional en Producción Vegetal Intensiva, Instituto Nacional de Investigación Agropecuaria (INIA), Camino al Terrible s/n, Salto 68033, Uruguay; msbres@inia.org.uy (M.S.); oblanco@inia.org.uy (O.B.); jlado@inia.org.uy (J.L.); 2 Unión de Productores y Exportadores Frutihortícolas del Uruguay (UPEFRUY), Rincón 487, Montevideo 11000, Uruguay; gerofernandez37@gmail.com; 3 Postcosecha, Estación Experimental Concordia, Instituto Nacional de Tecnología Agropecuaria (INTA), Ruta Provincial 22 y vías del Ferrocarril, Concordia 3200, Entre Ríos, Argentina; cocco.mariangeles@inta.gob.ar

**Keywords:** *Geotrichum citri-aurantii*, *Penicillium digitatum*, Proallium, food additives, biofungicides

## Abstract

The efficacy of natamycin (Fruitgard Nat 20) and Proallium (an extract of allium including propyl thiosulfinate oxide (PTSO)) against sour rot and green mold in mandarins was evaluated under controlled and commercial conditions. The study involved artificial inoculation of Nova, Tango, Orri, Afourer, Murcott, and Nules Clementine mandarins with isolates of *Penicillium digitatum* resistant to imazalil and pyrimethanil and an isolate of *Geotrichum citri-aurantii* susceptible to propiconazole fungicides. Under laboratory conditions, natamycin applied at 1500 µg mL^−1^ significantly reduced green mold by 61.2% in Orri and sour rot by 62.8% in Nova and 80% in Tango. Increasing the concentration to 2000 µg mL^−1^ further improved control of sour rot in Nova to 92.8%. In commercial trials, natamycin at 1500 µg mL^−1^ was ineffective on Afourer; however, 2000 µg mL^−1^ reduced sour rot by 39% on Nules Clementine. Proallium (12–16 µg mL^−1^ PTSO) applied under controlled conditions effectively reduced green mold by 33% in Nova and 31% in Nules Clementine, and sour rot by 19%, 41%, and 36% in Nules Clementine, Nova, and Afourer, respectively. Under commercial conditions, using the same dose of Proallium, there was a 51.5% reduction in the incidence of imazalil and pyrimethanil-resistant *P. digitatum* strains and a 36.5% reduction in sour rot. Both natamycin and PTSO showed promising results for managing green mold caused by fungicide-resistant strains, but further research is needed to optimize control of sour rot in mandarins.

## 1. Introduction

The genus *Citrus*, especially mandarins, comprises numerous species, cultivars, and hybrids, being a widely cultivated crop in demand worldwide [1,2,3,4]. This genetic diversity results in varying levels of susceptibility to several diseases, including green mold and sour rot [5,6,7,8,9]. Green mold and sour rot are caused by *Penicillium digitatum* and *Geotrichum citri-aurantii*, respectively, and are among the most economically significant postharvest citrus diseases worldwide [10,11]. Both diseases result in soft rot, which spoils the fruit and diminishes its marketability. Both are pathogens that take advantage of pre-existent wounds, and fruit can be infected in the field or during the harvest process. For many years, conventional synthetic fungicides have been applied during the packing-line process as curative treatments to prevent losses during storage and marketing [12].

Upon arrival at the packinghouse, fungicides are typically applied using drencher systems, immersion tanks, or in-line drenchers on the packing line and are often incorporated into wax coatings to protect the fruit [13,14]. Fungicides, including benomyl, imazalil, sodium orthophenylphenate (SOPP), thiabendazole (TBZ), pyrimethanil, azoxystrobin, and fludioxonil, have been extensively used to control green mold in citrus [6,15,16]. However, the continuous use of these fungicides has led to the selection and spread of resistant isolates, significantly diminishing their efficacy [17,18]. The control options for citrus sour rot are limited, but guazatine and propiconazole have been shown to be effective [19,20,21]. However, guazatine is not approved for use on citrus in the United States [19], and the European Union has set the maximum residue limit for propiconazole at 0.01 mg kg^−1^ [22], a level insufficient for effective control of *G. citri-aurantii.* This regulatory restriction complicates the use of propiconazole for sour rot management in the EU. Furthermore, the use of conventional fungicides faces increasing challenges due to health risks, consumer concerns [23], and the environmental impact of wastewater disposal from packinghouses [24]. This situation emphasizes the need to explore alternative methods that can effectively minimize or prevent fruit decay from harvest to consumption while adhering to regulatory and environmental standards.

Natamycin is a food additive derived from the fermentation of certain *Streptomyces* species [25]. In the United States, it is classified by the U.S. Food and Drug Administration (FDA) as Generally Recognized as Safe (GRAS), due to its exceptionally low toxicity to human and mammalian cells [26]. Its mechanism of action involves the inhibition of fungal growth through the disruption of ergosterol function within the fungal membrane [25]. Registered in the USA in 2016 as an effective biopesticide, natamycin is used to control *P. digitatum* and *G. citri-aurantii* [27]. A significant advantage of natamycin is its low risk of developing resistance, making it a valuable component of anti-resistance management strategies when used alongside conventional methods [28]. Furthermore, natamycin has demonstrated effective control of sour rot in lemons through a commercial strategy that excludes conventional fungicides [29].

Proallium (Domca S.A., Spain) is a commercially available food additive recommended for postharvest application to combat rot-causing pathogens in fruits and vegetables. The active ingredient, PTSO (propyl thiosulfinate oxide), is a bioactive compound derived from *Allium* species. Additionally, Food Coat (Domca S.A., Spain) is an edible coating formulated with sucrose esters of fatty acids and other food additives to enhance the adhesion of active ingredients. The combined use of Proallium and Food Coat has been shown to reduce postharvest pathogen rot in various fruits and vegetables [29]. The fungicidal efficacy of Proallium and Food Coat in controlling citrus sour rot and green mold has been evaluated in lemons and oranges, yielding promising results [29,30]. However, to our knowledge, there is currently no scientific evidence supporting the effectiveness of Proallium and Food Coat in controlling sour rot and green mold in mandarins.

This study aimed to evaluate the curative activity of the food additives natamycin (Fruitgard Nat_20) and PTSO (Proallium) in conjunction with Food Coat, focusing on their potential to reduce postharvest incidence of sour rot and green mold in mandarin fruit. Experiments were conducted under both laboratory and commercial conditions to assess the feasibility of integrating these compounds into a safe and effective postharvest control strategy against sour rot and green mold.

## 2. Materials and Methods

### 2.1. Fungal Isolates and Inoculum Preparation

The isolates of *P. digitatum* and *G. citri-aurantii* utilized in this study are detailed in Table 1. Isolates G0, S22, and R20 are part of the fungal collection at the Phytopathology Laboratory of INIA Salto Grande in Uruguay. The isolate A17 is preserved in the Postharvest Laboratory at INTA Concordia in Argentina.

A loop of the fungal suspension, cryopreserved at −80 °C, was recovered and added to potato dextrose agar (PDA) medium (Oxoid Ltd., Basingstoke, Hampshire, UK) and subsequently maintained on PDA at 4 °C for short-term storage. For inoculation, spores of *P. digitatum* isolates and arthrospores of *G. citri-aurantii* were harvested by adding 10 mL of 0.05 M KH_2_PO_4_ containing 0.01% (v/v) Triton X-100 to the surface of 7-day-old fungal cultures grown on Petri dishes with PDA medium. The resulting inoculum was filtered through two layers of cheesecloth, followed by dilution to an absorbance of 0.1 at 420 nm [33], achieving a final concentration of 1.0 × 10^6^ conidia mL^−1^. Additionally, cycloheximide (10% of a stock solution of 10 µg mL^−1^) was incorporated into the *G. citri-aurantii* inoculum to inhibit fruit wound healing [34].

### 2.2. Food Additives and Conventional Fungicides Used

**Food additives**: The experimental formulation, Fruitgard Nat 20, containing 20% natamycin, was supplied by Enzur (Montevideo, Uruguay). Proallium and an FoodCoat (composed of vegetable compounds) were provided by Vertisoles S.R.L (Concordia, Entre Rios, Argentina). Proallium is available in various formulations; in this experiment, we utilized Proallium FRD (with a PTSO concentration of 1200–1500 µg mL^−1^) and Proallium Brill (PTSO 600–800 µg mL^−1^). In Proallium Brill, the PTSO concentration is reduced by nearly 50% and the characteristic odor is notably attenuated.

**Conventional fungicides and wax**: The conventional fungicides used included fludioxonil (Scholar 23% SC), imazalil (Deccozil50 50% p/v or Fruitgard-IS 7.5 7.5% p/v), a premixed formulation of imazalil (20%) + pyrimethanil (20%) (Philabuster 400SC; Janssen PMP), a mixture of wax (18% solids) (Brillaqua UE-18) + imazalil (0.2% wp p/v), ortho-phenil phenol (Fruitgard-OPP-10 at 10% p/v), propiconazole (Fruitgard PZ-100 p.a 9.8% p/v), and 18% solid wax (Decco Citrashine).

### 2.3. Fruit

Fruits (free of any postharvest treatment) were harvested from commercial export orchards or withdrawn from packinghouses at different times, depending on the cultivars tested. According to the ripening period, the sequence was as follows: Nules Clementine (*C. clementina* hort. ex Tanaka) (March), Nova (hybrid *C. clementina* × tangelo) (May), Tango (*W. Murcott* induced mutation) (June), Afourer (hybrid Tangor) (July), Orri (hybrid Tangelo) (July), Murcott (origin unknown) (August). Fruits were selected for uniform size, color, and absence of lesions. In the laboratory, various cultivars of mandarins were used depending on the trial (see Table 2 and Table 3). For the trials under commercial conditions, Nules Clementine, Murcott, and Afourer were utilized.

Before each experiment, the fruits were washed and superficially disinfected by immersion for one minute in a solution containing 0.2 µg mL^−1^ of active chlorine, followed by rinsing with tap water. Fruits inoculated with *G. citri-aurantii* were pre-treated by immersion for one minute in a 100 µg mL^−1^ imazalil solution to minimize interference from infection by *Penicillium* spp. The treated fruits were then placed in disinfected plastic boxes (dimensions: 50.5 cm × 33.5 cm × 28 cm) and kept in a clean room for 24 h before the trials commenced.

### 2.4. Fruit Fungal Inoculation

The disinfected and dried fruits were randomized and placed into a 23-cavity cardboard container. One inoculation per fruit was performed at the equatorial zone using a stainless-steel wounding tool, measuring 2 mm diameter and 1 mm in depth, which had been previously immersed in the inoculum suspension, as described by Eckert and Brown [33]. The inoculum density was maintained at 1 × 10 ^6^ spores mL^−1^. To maintain humidity, containers with fruit inoculated with the G0 isolate were covered with plastic bags that had been sprayed with sterile distilled water. The inoculation procedure was conducted 18 to 20 h prior to the application of the respective treatments to evaluate the curative properties of the alternative products.

### 2.5. Sensitivity to Natamycin of Penicillium digitatum Isolates Susceptible (S22) and Resistant (R20) to Imazalil

The effective concentration inhibiting 50% growth (EC_50_) and the minimum inhibitory concentration (MIC) of *P. digitatum* isolates S22 and R20 were determined following the modified procedures outlined by Hadaeck and Greger [35]. A stock solution of natamycin at a concentration of 32 µg mL^−1^ was prepared with sterile distilled water, employing technical-grade natamycin (95.5%) sourced from Enzur S.A. (Montevideo, Uruguay). The concentration of natamycin was quantified via high-performance liquid chromatography (HPLC) using a Restek Ultra C18 column (150 mm × 4.5 mm, 5 µm particle size) with a mobile phase consisting of acetonitrile and water (40:60) at a flow rate of 0.5 mL min^−1^.

Wells in 6 columns of a 96-well plate were filled with 100 µL of potato broth (35 g L^−1^). A 100 µL aliquot of the natamycin stock solution was added to each well in the first column, followed by serial two-fold dilutions across columns one to five, using a 100 µL multichannel pipette. Subsequently, 100 µL of inoculum suspension containing 1 × 10^6^ spores mL^−1^) was dispensed into all wells across the columns. This procedure resulted in final natamycin concentrations ranging from 0–8 µg mL^−1^.

### 2.6. Curative Activity of Natamycin and PTSO in Inoculated Mandarin Fruit

#### Controlled Conditions Treatments (Immersion)

The effective concentration of natamycin for controlling the *P. digitatum* isolate R20 and the *G. citri-aurantii* isolate G0 was determined through dipping treatments on artificially inoculated fruit (Table 2).

For all treatments, fruits were immersed in 20 L of each aqueous solution at room temperature (20 to 23 °C) for 60 s. Immediately following treatment, the fruits were randomized and repacked into 23-cavity cardboard containers, which were then placed in plastic boxes measuring 52 cm × 36 cm × 16 cm. These boxes were maintained in an environment at 25 ± 2 °C for a duration of 7 days. The boxes containing fruit inoculated with *G. citri-aurantii* remained covered with polyethylene bags throughout the experimental period.

To ensure conditions of high humidity within the boxes, sterile distilled water was sprinkled there at the beginning of the experiment. The incidence of decay was calculated as the ratio of the number of rotten fruits in the inoculation zone to the total number of inoculated fruits, multiplied by 100.

For all trials conducted under laboratory conditions, three replicates of 20–30 fruits each were used for the green mold assessments, while six replicates of 20–23 fruits each were employed for the sour rot evaluations. In all trials, control fruits were treated with water.

### 2.7. Commercial Treatments

#### 2.7.1. Natamycin (Commercial Drencher)

The application of natamycin for the control of green mold and sour rot was conducted via a commercial chain drencher at a packinghouse located in Salto, Uruguay. Inoculated fruits were distributed inside net bags placed on top of a bin filled with additional fruit. This bin was then positioned atop a three-bin tower. The duration of the natamycin application was 30 s.

Concentrations of natamycin at 1500, 2000, and 2500 µg mL^−1^ were evaluated for the control of sour rot in Afourer and Nules Clementine cultivars. The efficacy of natamycin at a concentration of 2000 µg mL^−1^ was specifically assessed for control of green mold in the Nules Clementine mandarins.

The numbers of fruits and replicates used in these trials were identical to those of the treatments under controlled conditions.

#### 2.7.2. Proallium + Food Coat (Commercial and Experimental Packing Line)

The efficacy of Proallium Brill (12–16 µg mL^−1^ PTSO) for control of sour rot was evaluated on an experimental packing line in INIA Salto Grande, Uruguay, using fruit artificially inoculated with isolate G0 (Table 3). A total of 120 Afourer mandarin fruits were utilized for each treatment.

The control of green mold in mandarin fruit (Murcott) was assessed under commercial processing conditions in a packinghouse in Concordia, Argentina. The fruits were inoculated with isolate A17, which is resistant to the fungicides imazalil and pyrimethanil, as previously described. Twenty hours post-inoculation, the fruits were processed alongside commercially packed lemons. The processing involved sequential washing with detergent, rising with water, and treatment with either conventional fungicides (imazalil 1.2 µr mL^−1^ and fludioxonil 0.6 µg mL^−1^) or the experimental additive Proallium FRD (12–16 µg mL^−1^).

These treatments were applied with a flooder system, calibrated to 2 L per ton of citrus fruit, over rotating brushes. Subsequently, the fruits were dried and waxed. An absolute control was established using fruits that were inoculated but treated with water. Three replicates of 30 fruits were utilized for each treatment. The fruits were stored in telescopic boxes at room temperature (20–25 °C) for 14 days, after which the incidence of green mold was evaluated.

### 2.8. Statistical Analysis

Different libraries from R software v.3.6.1 were used for the statistical analyses. The EC_50_ was estimated through regression analysis using the “drc” package. The dose–response relationship was modeled with a four-parameter curve (lower and upper asymptote, slope, and the effective concentration for 50% growth inhibition). Symmetric (log-logistic) and asymmetric (Weibull) dose–response models were compared using the Akaike information criterion (AIC).

In all experiments with natamycin and Proallium for controlling green mold and sour rot, the number of diseased (decayed) fruits was recorded for each replicate, and the incidence of pathogens was calculated as the percentage of diseased fruit (diseased fruit/total fruit x 100). A generalized linear model with binomial error was used to assess the relationship between disease incidence and treatments. Mean comparisons among treatments were conducted using the Tukey procedure (*p*-value ≤ 0.05). The reduction in disease incidence was calculated as follows:

$$\mathrm{Reduction}=1-\frac{\mathrm{Treatment}\;\mathrm{incidence}}{\mathrm{Control}\;\mathrm{incidence}}*100.$$

## 3. Results

### 3.1. Natamycin Sensitivity of Penicillium digitatum Isolates S22 and R20 (EC_50_ and MIC)

Natamycin effectively inhibited the growth of both tested *P. digitatum* isolates. The EC_50_ values ranged from 0.518 to 0.570 µg mL^−1^ with a mean of 0.544 ± 0.013 µg mL^−1^ for isolate R20, and from 0.564 to 0.638 µg mL^−1^ with a mean of 0.600 ± 0.018 µg mL^−1^ for isolate S22. Based on the AIC, the Weibull distribution (asymmetric around the upper and lower asymptotes) provided the best fit for the data. The minimum inhibitory concentrations were determined to be 0.6 µg mL^−1^ for isolate R20 and 1 µg mL^−1^ for isolate S22.

### 3.2. Controlled Conditions Treatments (Immersion)

#### 3.2.1. Efficacy of Natamycin Against Sour Rot and Green Mold Decay

**Figure 1:**
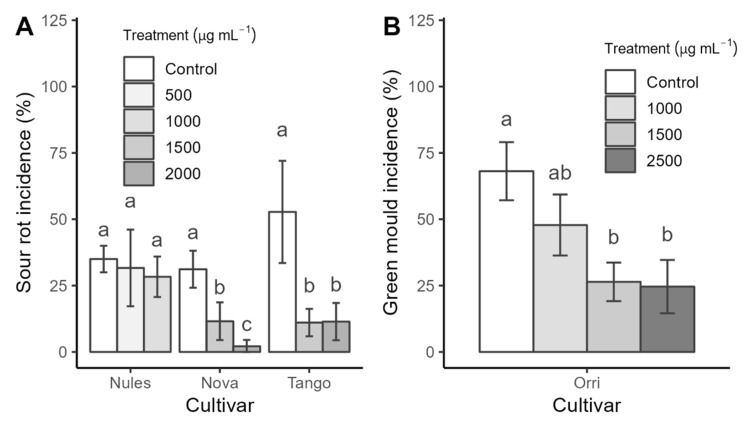
Efficacy of different natamycin concentrations in controlling sour rot in Nules Clementine, Nova, and Tango cultivars (**A**) and green mold in Orri cultivar (**B**). Data are presented as means (± standard error). Treatments with different letters indicate significant differences (Tukey *p*-value ≤ 0.05). References: control treatment with water; 500, 1000, 1500, 2000, and 2500 represent natamycin concentrations.

The effectiveness of natamycin in controlling sour rot on mandarins varied across the cultivars tested (Figure 1A). In Nules Clementine, application of 1000 µg mL^−1^ or lower doses did not provide significant disease control (Figure 1A), prompting an increase in concentration to 1500 and 2000 µg mL^−1^ for subsequent trials with Nova and Tango mandarins (Figure 1A). In Tango, natamycin applications (1500 and 2000 µg mL^−1^) reduced sour rot by approximately 80%. In contrast, sour rot control in Nova was concentration-dependent, registering a reduction in incidence of pathogens of 62.8% after 1500 µg mL^−1^ application and 92.9% after application of 2000 µg mL^−1^ (Figure 1A).

Similar to the results observed for control of sour rot, the application of 1000 µg mL^−1^ of natamycin did not effectively reduce decay caused by the *P. digitatum* isolate resistant to imazalil. However, a significant reduction in decay (63.24%) was achieved with 1500 µg mL^−1^. Increasing the concentration to 2500 µg mL^−1^ did not further enhance the reduction in decay (Figure 1B).

#### 3.2.2. Efficacy of Proallium + Food Coat Against Sour Rot and Green Mold Decay

Proallium Brill (12–16 µg mL^−1^ of PTSO) effectively controlled both green mold and sour rot in Nules Clementine, Nova, and Afourer cultivars (Table 4). Increasing the product concentration to 24–32 µg mL^−1^ enhanced the control of green mold, resulting in an incidence reduction exceeding 70% for Nules Clementine and nearly 90% for Nova. However, the reduction in incidence of sour rot remained below 50% across all conditions (Table 4).

### 3.3. Commercial Conditions Trials

#### 3.3.1. Evaluation of Natamycin Efficacy in a Commercial Drencher

**Figure 2:**
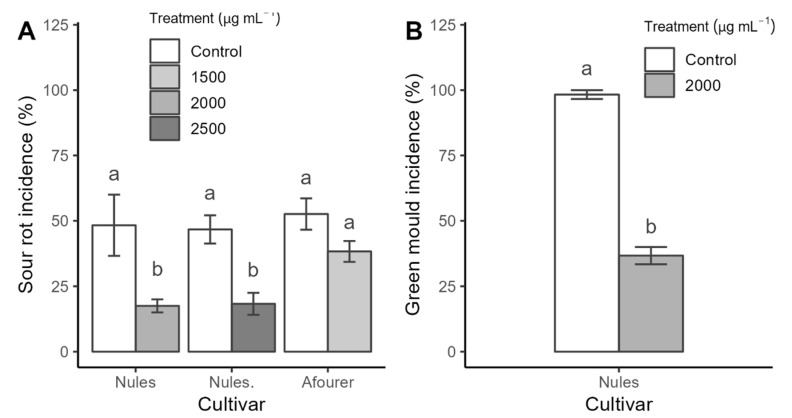
Incidence of sour rot (**A**) caused by *G. citri-aurantii* (isolate G0, susceptible to propiconazole) and green mold (**B**) caused by *P. digitatum* (isolate R20, resistant to imazalil) on Afourer or Nules Clementine cultivars. Means (± standard error) with different letters between treatments indicate significant differences (Tukey, *p*-value ≤ 0.05). References: control treatment with water; 1500, 2000, and 2500 represent natamycin concentrations.

Based on previous results, concentrations of natamycin of 1500 µg mL^−1^ and higher were tested via treatment with a commercial drencher. The application of 2000 µg mL^−1^ significantly reduced the incidence of both sour rot (Figure 2A) and green mold (Figure 2B) in Nules Clementine. However, the use of 1500 µg mL^−1^ was effective in controlling sour rot in Nules Clementine but not in the Afourer cultivar (Figure 2A).

#### 3.3.2. Evaluation of Proallium + Food Coat in Experimental and Commercial Packing-Line

**Figure 3:**
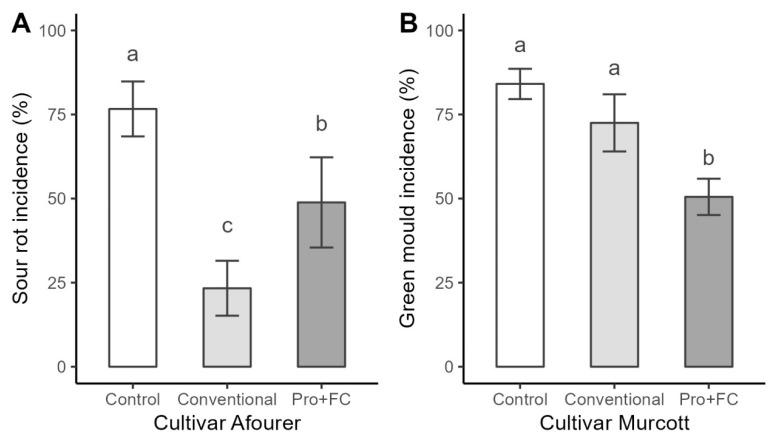
Incidence of sour rot in (**A**) and green mold (**B**) in artificially inoculated fruit. Different letters between treatments indicate significant differences (Tukey, *p*-value ≤ 0.05). Control: fungicide replaced by water; Conventional: propiconazole (0.98 µg mL^−1^) (**A**), imazalil (1.2 µg mL^−1^) and fludioxonil (0.6 µg mL^−1^) (**B**); Pro + FC: Proallium Brill (PTSO 12–16 µg mL^−1^) + Food Coat (2%) (**A**), Proallium FDR (PTSO 12–16 µg mL^−1^) + Food Coat (2%) (**B**).

In semi-commercial packing-line conditions, significant differences were observed among treatments for sour rot control. The highest reduction in incidence of sour rot (69.5%) was achieved with conventional treatment using propiconazole (1%), followed by Proallium Brill (PTSO 12–16 µg mL^−1^) + Food Coat (2%), which reduced incidence by 36.5% (Figure 3A).

Conventional fungicide treatments were ineffective against the imazalil and pyrimethanil-resistant isolate of *P. digitatum*. The incidence of green mold was 73% ± 8, which was not significantly different from the control (84% ± 4). However, the Proallium FRD (PTSO 12–16 µg mL^−1^) + Food Coat (2%) treatment significantly reduced the incidence of green mold to 51.5% ± 5 (Figure 3B).

## 4. Discussion

Our findings demonstrate that the food additive natamycin provided effective control of both sour rot and green mold across various mandarin cultivars in laboratory and commercial settings. Natamycin emerged as a promising alternative for managing *P. digitatum* isolates resistant to imazalil fungicides. Furthermore, natamycin has been used effectively to control sour rot and green mold in lemons and oranges [20,29,36], positioning it as a valuable tool for inclusion in strategies aimed at minimizing pathogen resistance in citrus crops. While previous studies have highlighted natamycin’s efficacy in controlling these diseases in Tango and Dancy mandarins [28,36], this is the first report demonstrating successful control of sour rot and green mold in Nules Clementine mandarins using a drencher treatment under commercial conditions.

Minimum inhibitory concentrations (MICs) of natamycin have been reported ranging from 0.5 to 6 µg mL^−1^, with some species requiring 10 to 25 µg mL^−1^ [37]. Our findings align with previously reported values for both *P. digitatum* and *G. citri-aurantii* [28,29,38]. Based on these similarities between isolates of different origins, we hypothesize that natamycin in the range between 500 and 1000 µg mL^−1^, as recommended by Adaskaveg et al. [27], could be effective for the control of sour rot and green mold in mandarins. These doses were successful in controlling *G. citri aurantii* on lemons in trials conducted under varying conditions and with different isolates [27,29]. The efficacy of natamycin for controlling sour rot appears to be higher in grapefruit and lemon than in orange or mandarin [36]. However, in our experiments, even with dip application, these doses were ineffective, and significant reductions in sour rot and green mold decay were observed only with concentrations of 1500 µg mL^−1^ or higher in most cultivars except Afourer (under commercial conditions). These variations may be attributable to differences in natamycin sources, inoculation, or treatment methods, or potential differences in the pathogen-additive interaction among isolates. Moreover, under the same experimental conditions, we observed that increasing the natamycin concentration significantly improved disease control in one cultivar (Nova), whereas in others (Nules Clementine and Tango), it did not.

With respect to Proallium, this food additive, in combination with FoodCoat and applied via immersion or on a commercial citrus packing line, demonstrated effective control of sour rot in Nova and Afourer mandarins, as well as green mold in Orri and Murcott, compared with untreated fruit. Similar results have been observed for lemons and oranges [29,30]. The significant efficacy of Proallium in controlling *P. digitatum* isolates resistant to the fungicides imazalil and pyrimethanil suggests that PTSO could be recommended as part of an anti-resistance management strategy for post-harvest treatment.

However, the efficacy of sour rot control was affected by the type of mandarin cultivar. Proallium Brill (PTSO 12–16 µg mL^−1^) achieved approximately 40% disease reduction for Nova and Afourer cultiuvars. In contrast, for Nules Clementine mandarins, similar control was achieved only by doubling the concentration of PTSO. Proallium refers to a range of formulations whose active ingredient, PTSO, is derived from extracts of plants in the Allium genus. As plant extracts are complex mixtures, the composition may vary based on the source of the plant material and the extraction method [39]. This variability in PTSO concentration can affect the reproducibility of results. In the present study, both Proallium Brill and Proallium FRD, when used at the same concentration of PTSO, resulted in significant control of sour rot and green mold.

Nazerian and Alian [8] highlighted that the incidence of sour rot decay varies significantly among mandarin species and hybrids. To date, there has been limited research on the underlying causes of this variability, but it may be attributed to genetic differences between cultivars. Early studies by Suprapta et al. [40] revealed that citrus-specific isolates of *G. candidum* infected lemons and Satsuma mandarins, but not all isolates were capable of infecting oranges. The authors suggested that those differences may have been due to variations in the chemical composition and physical structure of the fruit peel across citrus species.

Badouin and Eckert [41] conducted an extensive study to identify factors associated with susceptibility of lemon fruit to sour rot. Their research indicated significant variability among individual lemons harvested from the same plot at the same time. This heterogeneity could not be linked to specific trees within the grove or to fruit position on the tree. Additionally, they reported considerable differences in susceptibility to *G. citrus aurantii* among fruits from different groves, suggesting that environmental factors and agronomic practices may play a significant role. Ultimately, their study associated fruits’ susceptibility to sour rot with the physiological age of the peel and its water content, rather than with cultivar [41].

Further research is needed to explore the influence of physicochemical, environmental, genetic, and agronomic factors on the behavior of sour rot in mandarins, in order to optimize disease management strategies.

## 5. Conclusions

Natamycin demonstrated effective control of sour rot and green mold across various mandarin cultivars in both the laboratory and commercial drencher trials (1500 and 2000 µg mL^−1^), proving it to be a viable alternative for enhancing the control of *P. digitatum* isolates resistant to imazalil and phyrimethanil.

Proallium Brill emerged as a promising product for controlling resistant *Penicillium digitatum* isolates and can serve as a valuable complement for the integrated postharvest management of sour rot in mandarins, achieving approximately 50% disease control under both laboratory and semi-commercial or commercial conditions.

## Figures and Tables

**Table 1:** Isolates of *Geotrichum citri-aurantii* and *Penicillium digitatum* utilized for artificial inoculations of fruit in various trials.

Isolate Code	Species	Characteristic
G0	*Geotrichum citri-aurantii*	Susceptible to propiconazole [31]
S22	*Penicillium digitatum*	Susceptible to imazalil [20]
R20	*Penicillium digitatum*	Resistant to imazalil [20]
A17	*Penicillium digitatum*	Resistant to imazalil and pyrimethanil [32]

**Table 2:** Evaluated natamycin concentrations in mandarin cultivars for effective control of *G. citri-aurantii* and *P. digitatum*. Fruits were artificially inoculated with the isolates G0 (*G. citri-aurantii*) or R20 (*P. digitatum*).

Trial	Mandarin Cultivar	Natamicyn (µg mL^−1^)	Pathogen
1	Nules Clementine (*Citrus clementina* hort. Ex Tanaka)	500, 1000	*G. citri-aurantii*
2	Nova (*Citrus reticulata* (Blanco))	1500, 2000, 2500	*G. citri-aurantii*
3	Tango (*Citrus reticulata* (Blanco))	1500, 2000, 2500	*G. citri-aurantii*
4	Orri (*Citrus reticulata* (Blanco)	1000, 1500, 2500	*P. digitatum*

**Table 3:** Evaluation of sour rot control using Proallium on an experimental packing line. Afourer mandarin fruits were artificially inoculated with the G0 isolate (*G. citri-aurantii*).

	Packing-Line Sequential Treatments		
Treatment	Washed	Fungicide^,^	Coating
Proallium	Neutral soap (10%)	Proallium Brill ^1^ (12–16 µg mL^−1^ PTSO)	Wax + imazalil (2%)
Control+	Neutral soap (10%)	Water	Wax + imazalil (2%)
Control−	SOPP (10%)	Propiconazole (98 µg mL^−1^)	Wax + imazalil (2%)

^1^ = Proallium Brill was applied in combination with Food Coat (2%).

**Table 4:** Sour rot and green mold incidence (%) or incidence reduction (%) on Nules Clementine, Nova and Afourer cultivars. Fruits were immersed for 1 min in Proallium or water (control).

	Nules Clementine				Nova				Afourer	
	Sour Rot		Green Mold		Sour Rot		Green Mold		Sour Rot	
Treatment	Inc. (%)	Red2. (%)	Inc. (%)	Red. (%)	Inc. (%)	Red. (%)	Inc. (%)	Red. (%)	Inc. (%)	Red. (%)
Control	88.2 a	-	88.3 a	-	45.4 a	-	100 a	-	53.07 a	
Proallium Brill (PTSO 12–16 µg mL^−1^)	71.3 b	19.1	61 b	30.9	26.8 b	40.9	66.7 b	33.3	33.92 b	36.1
Control	88.3 a	-	100 a	-	61.1 a	-	68.9 a	-	53.07 a	
Proallium Brill (PTSO 24–32 µg mL^−1^)	52.1 b	41.0	29.8 b	70.2	43.5 b	28.8	8.9 b	87.1	27.30 b	48.6

Different letters indicate significant differences between treatments (Tukey, *p*-value ≤ 0.05). Proallium Brill was applied in mix with Food Coat (2%). Inc, incidence; Red, disease reduction.

## Data Availability

The raw data supporting the conclusions of this article will be made available by the authors on request.
